# Global research trends in necrotizing pancreatitis: a bibliometric analysis from 2013 to 2024

**DOI:** 10.3389/fmed.2025.1515418

**Published:** 2025-01-22

**Authors:** Gulnur G. Gainollina, Murat K. Jakanov, Bazylbek S. Zhakiev, Uteugaly G. Karsakbayev, Kairat R. Taishibayev, Bulat A. Kurmanbayev

**Affiliations:** ^1^Department of General Surgery, West Kazakhstan Marat Ospanov Medical University, Aktobe, Kazakhstan; ^2^Department of Surgical Diseases No. 2, West Kazakhstan Marat Ospanov Medical University, Aktobe, Kazakhstan

**Keywords:** acute pancreatitis, necrotizing pancreatitis, pancreatic necrosis, mortality, bibliometric analysis

## Abstract

**Background:**

This study aims to analyzing scientific publications related to necrotizing pancreatitis and its mortality, identifying key areas and trends, and determining the leading research institutions, authors, countries, and journals actively working in this field.

**Methods:**

The Web of Science and Scopus databases were searched for articles on NP published between January 1, 2013, and April 22, 2024. Articles published before 2013, conference abstracts, and case reports were excluded. The articles were assessed based on various metrics, including the number of citations, publication dates, countries of origin, institutions, journals, and authors.

**Results:**

A total of 929 articles were identified, of which 251 were deemed suitable for analysis after duplicates were removed. China contributed the most articles, followed by the United States and India. The most frequent publications appeared in specialized journals such as “Pancreatology” and “Journal of Gastrointestinal Surgery.” The primary research institutions were universities and medical centers. The highest-impact articles focused on minimally invasive treatment methods for NP. There has been a growing body of research in NP over the past decade, particularly in China and the United States.

**Conclusion:**

Despite advancements in medical science, the mortality rate associated with pancreatic necrosis remains high. This highlights the continued challenge in effectively addressing complications of acute pancreatitis. Researchers worldwide are actively exploring alternative therapeutic approaches to mitigate these complications and improve patient outcomes.

## 1 Introduction

Acute pancreatitis (AP) is a widespread condition affecting the pancreas and is the primary cause of hospitalization in surgical wards among gastrointestinal diseases (1). According to a systematic review and meta-analysis by Iannuzzi et al. (2) the incidence of acute pancreatitis has been increasing annually from 1961 to 2016. The overall incidence has risen by 3.07% per year. This trend has been observed in most regions worldwide, including North America, Europe, South America, and Oceania, while in Asia, the rates have remained relatively stable (2).

Zilio et al. (3) conducted a comprehensive analysis of publications to identify the etiology of acute pancreatitis (AP), including data from 46 studies involving a total of 2,341,007 patients across 36 countries. The global estimates for the distribution of etiologies were 42% (95% CI: 39–44%) for biliary, 21% (95% CI: 17–25%) for alcoholic, and 18% (95% CI: 15–22%) for idiopathic (3). Less commonly, these include tumors, trauma, hypertriglyceridemia, and the use of certain medications, such as azathioprine, furosemide, and corticosteroids (4, 5). Additionally, AP can develop as a result of iatrogenic injuries, particularly following endoscopic retrograde cholangiopancreatography or surgical procedures (6).

Approximately 80% of acute pancreatitis (AP) cases present in a mild edematous form, while around 20% progress to severe or complicated pancreatitis, which is characterized by early or delayed systemic and local complications (7). In severe cases, up to 10.5% may require surgical intervention, and up to 40% may die during hospitalization (8). Overall, mortality in severe AP can reach 50%, contrasting with an overall mortality rate of 2–5% for all forms of AP (9). This increased risk of mortality and complications is often associated with the development of pancreatic necrosis, which is frequently accompanied by infectious complications (10, 11). Mortality rates in these cases can range from 15 to 39% (12). Specifically, in North America and Europe, the mortality rates vary between 18 and 20% (13), while in China, they range from 8 to 39% (14, 15) and in Japan, the rates fluctuate between 10 and 26% (16). Pancreatic necrosis may involve the pancreatic parenchyma, the peripancreatic tissues, or both regions simultaneously (17).

Surgical intervention is usually required for the management of infected pancreatic necrosis (18), and, less frequently, for sterile necrosis when it leads to gastric, duodenal, or biliary obstruction (12, 19). For many years, open surgical necrosectomy was the standard treatment for infected necrotizing pancreatitis, despite its high complication rate and significant mortality (18). However, in the past two decades, there has been a shift toward minimally invasive techniques, such as percutaneous abscess drainage, endoscopic transgastric necrosectomy (ETN), laparoscopy, and video-assisted retroperitoneal debridement (VARD) (20). Mehta et al. (20) and van Baal et al. (21), in their study, highlight the significance of percutaneous drainage as a primary method for effectively eliminating abscesses and minimizing the need for open surgical interventions (20, 21). Similarly, Tan et al. (22) and Mathew et al. (23) explore laparoscopic necrosectomy, which offers significant advantages by minimizing tissue trauma. Additionally, Revoredo Rego et al. (24) investigate VARD a technique that ensures precise access to necrotic tissue while reducing the invasiveness of the procedure (24). Moreover, Bakker et al. (25) compare ETN with conventional surgical necrosectomy. Collectively, these studies highlight the efficacy of minimally invasive techniques in enhancing clinical outcomes, reducing hospitalization duration, and promoting faster recovery.

In recent years, the focus of bibliometric research has shifted significantly toward the study of minimally invasive surgery, pancreatic cystic disease, and oncological interventions, which have emerged as dominant topics in scientific publications (26–28). Acute pancreatitis has also become a focus of attention among researchers. Bibliometric analyses in this field have predominantly utilized the Web of Science (WoS) database and CiteSpace software to identify trends and patterns (29, 30). To broaden the scope and ensure a more thorough evaluation, our study includes both the WoS and Scopus databases, we employed RStudio for advanced data processing and visualization. To fill the gap in the existing literature, we conducted a bibliometric analysis aimed at analyzing scientific publications related to necrotizing pancreatitis and its mortality, identifying key areas and trends, and determining the leading research institutions, authors, countries, and journals actively working in this field.

## 2 Materials and methods

### 2.1 Search strategy and data collection

**Figure 1:**
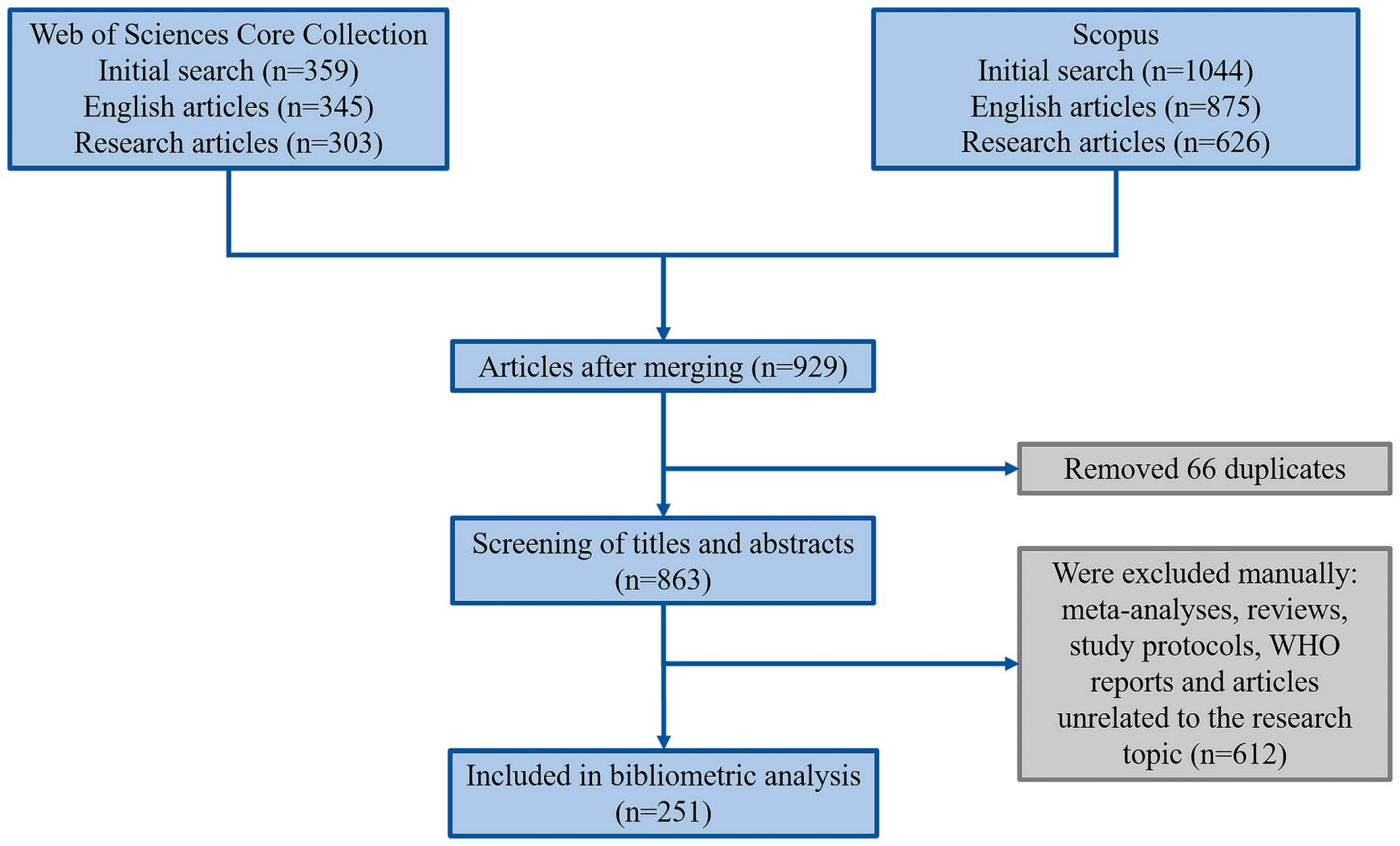
Article selection flowchart.

The literature search was conducted in two databases, Web of Science and Scopus, covering the period from January 1, 2013, to April 22, 2024. Research articles written in English were selected. Case reports, conference abstracts, and editorial materials were excluded from the analysis. A combination of keywords (“necrotizing pancreatitis” and “mortality”) and their synonyms were used, employing Boolean operators (AND, OR). Information regarding authors, titles, sources, sponsors, abstracts, document types, cited references, and keywords was retrieved in txt format for Web of Science and in BibTeX format for Scopus for further analysis. The obtained data were merged using RStudio. The result was a single file in xlsx format. The detailed search strategy is demonstrated in Figure 1.

### 2.2 Study selection and data extraction

The titles, abstracts, and full texts of the articles were thoroughly reviewed by two authors (GGG and MKJ) to determine their relevance to the objectives of the study. In case of disagreements, a third author was consulted to help resolve any discrepancies.

### 2.3 Visualization and statistical tools

Data processing and analysis were performed using the Bibliometrix package (version 3.2.1^1^) in the RStudio environment (version 4.1.2). The Biblioshiny web interface allowed for the creation of various visualizations, bibliometric mapping, and statistical calculations.

## 3 Results

### 3.1 Summary of the papers

**Figure 2:**
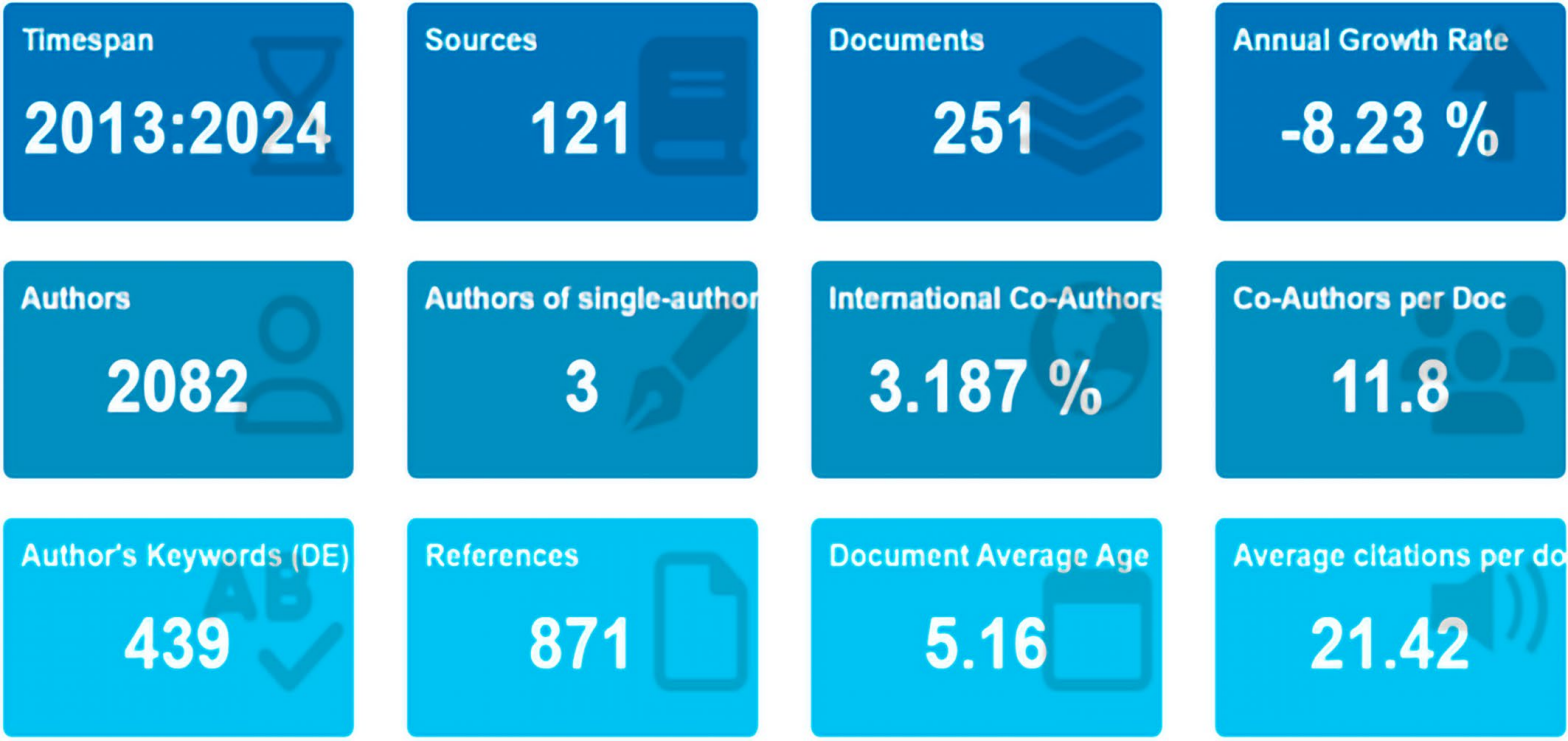
Main information on the bibliometric analysis of publications on necrotizing pancreatitis.

This bibliometric analysis includes 251 documents from 121 journals over the period from 2013 to 2024 (Figure 2). The mean age of the documents was 5.16 years, with an average citation count of 21.42 per document. Among the 2,082 authors, only 3 produced a document independently, while the mean number of co-authors per document was 11.8. A total of 1,838 keywords were identified, with 439 occurring most frequently. The analysis also revealed an annual growth rate of −8.23%.

### 3.2 Annual analysis of publication

**Figure 3:**
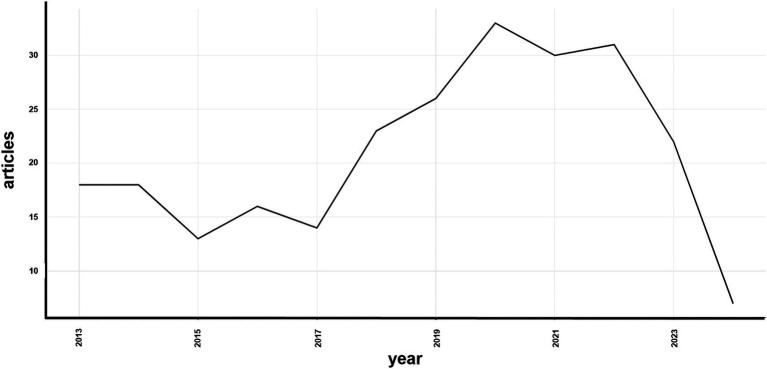
Annual scientific production.

The annual scientific publication trends, shown in Figure 3, indicate fluctuations from 2013 to 2024. From 2013 to 2017, the number of publications remained relatively stable with minor variations. However, from 2018 to 2021, a noticeable increase occurred, peaking in 2020 with 33 articles. A slight decrease in publications was observed in 2023, with 23 articles published.

### 3.3 The most productive country

**Figure 4:**
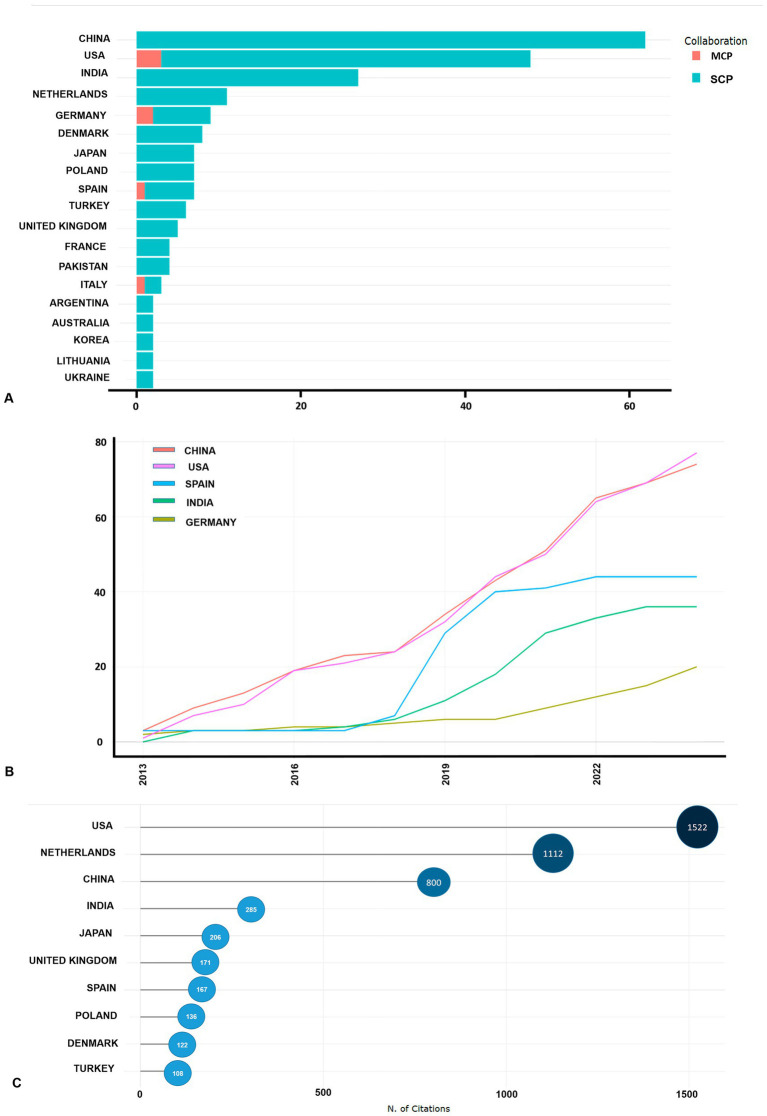
Scientific collaborations within countries and international scientific research projects. SCP, Single country publications; MCP, Multiple country publications **(A)**, countries’ production over time **(B)**, top 10 most cited countries **(C)**.

The global research landscape on necrotizing pancreatitis (NP) spans 35 countries, with the most prolific contributors being China (62 publications), the United States (45 publications), and India (27 publications). Additionally, the United States, Germany, and Spain are involved in international collaborations (Figure 4A). Notably, China and the United States have demonstrated the most substantial growth in publication output, with their contributions increasing from 3 and 1 in 2013 to 74 and 77 in 2024, respectively (Figure 4B). Other countries, such as Germany, Japan, and India, have shown steady growth, while Spain experienced a significant surge in publications from 2018, peaking in 2020. The United States and the Netherlands lead in citations with 1,522 and 1,112, respectively, followed by China and India with 800 and 285 citations, respectively (Figure 4C).

### 3.4 Core journals and references

The largest number of articles on necrotizing pancreatitis was published in *Pancreatology* (24 articles), followed by the *Journal of Gastrointestinal Surgery* (13 articles) and *Pancreas* (11 articles) (Table 1). The journal with the highest total citation count was *Annals of Surgery*, with 500 citations, a CiteScore of 16.2, and classification in the Q1 JCR category. *Pancreatology* ranked second in total citations (424), with a CiteScore of 6.6, and is categorized in Q2. The *Journal of Gastrointestinal Surgery* and *Pancreas* are also positioned in the first and second quartiles, respectively, and demonstrate high citation rates and impact indexes. Conversely, journals such as the *American Surgeon* and the *ANZ Journal of Surgery* have lower citation metrics and are classified in Q3.

**Table 1:** Top 10 journals with the most citied articles.

Rank	Journal	Number of articles	Total citation	2024 JCR category quartile	CiteScore	h-index	g-index	m-index
1	Pancreatology	24	424	Q2	6.6	13	20	1.083
2	Journal of Gastrointestinal Surgery	13	202	Q1	5.2	9	13	0.750
3	Pancreas	11	108	Q2	4.1	5	10	0.417
4	Annals of surgery	10	500	Q1	16.2	8	10	0.727
5	Surgical endoscopy	7	87	Q1	6.0	6	7	0.500
6	BMC Gastroenterology	6	124	Q3	3.9	3	6	0.250
7	Digestive Diseases and Sciences	6	57	Q2	6.2	4	6	0.800
8	ANZ Journal of Surgery	5	31	Q2	2.2	3	5	0.273
9	Scandinavian Journal of Gastroenterology	5	79	Q3	3.7	4	5	0.333
10	American Surgeon	4	11	Q3	1.5	2	3	0.333

Table 2 highlights 10 notable scientific articles published across various journals, detailing their DOI, total citations, average citations per year, and normalized citation counts. The article by Van Brunschot et al. (31) in *Lancet* recorded the highest total citations (466) and a normalized citation count of 10.35. Additionally, the article by Baron et al. (32) in *Gastroenterology* achieved the highest average citations per year (78.60) and a normalized citation count of 15.93. Other significant contributions include works by Trikudanathan et al. (45, 46), which also demonstrated high citation rates.

**Table 2:** Top 10 most cited articles.

Rank	Title of the article	Authors	DOI	Year	Journal	Total citations	TC per year
1	Endoscopic or surgical step-up approach for infected necrotising pancreatitis: a multicenter randomized trial	Van Brunschоt S et al.	https://doi.org/10.1016/S0140-6736(17)32404-2	2018	Lancet	466	66.57
2	American Gastroenterological Association Clinical Practice Update: Management of Pancreatic Necrosis	Baron T et al.	https://doi.org/10.1053/j.gastro.2019.07.064	2020	Gastroenterology	393	78.60
3	Current Concepts in Severe Acute and Necrotizing Pancreatitis: An Evidence-Based Approach	Trikudanathan G et al.	https://doi.org/10.1053/j.gastro.2019.01.269	2019	Gastroenterology	212	35.33
4	An Endoscopic Transluminal Approach, Compared With Minimally Invasive Surgery, Reduces Complications and Costs for Patients With Necrotizing Pancreatitis	Bang JY et al.	https://doi.org/10.1053/j.gastro.2018.11.031	2019	Gastroenterology	197	32.83
5	Minimally invasive and endoscopic versus open necrosectomy for necrotising pancreatitis: a pooled analysis of individual data for 1980 patients	Van Brunschоt S et al.	https://doi.org/10.1136/gutjnl-2016-313341	2018	GUT	141	20.14
6	Dual-modality drainage of infected and symptomatic walled-off pancreatic necrosis: long-term clinical outcomes	Ross AS et al.	https://doi.org/10.1016/j.gie.2013.10.014	2014	Gastrointestendoscopy	120	10.91
7	Japanese multicenter experience of endoscopic necrosectomy for infected walled-off pancreatic necrosis: The JENIPaN study	Yasuda I et al.	https://doi.org/10.1055/s-0033-1344027	2013	Endoscopy	119	9.92
8	Immediate versus Postponed Intervention for Infected Necrotizing Pancreatitis	Boxhoorn L et al.	https://doi.org/10.1056/NEJMoa2100826	2021	New England Journal Medicine	111	27.75
9	The Role of Organ Failure and Infection in Necrotizing Pancreatitis A Prospective Study	Guo Q et al.	https://doi.org/10.1097/SLA.0000000000000264	2014	Annals of Surgery	109	9.91
10	Early (<4 Weeks) versus Standard (≥4 Weeks) Endoscopically Centered Step-Up Interventions for Necrotizing Pancreatitis	Trikudanathan G et al.	https://doi.org/10.1038/s41395-018-0232-3	2018	American Journal Gastroenterology	107	15.29

### 3.5 The most productive institutions and authors

Table 3 highlights the 10 most productive institutions contributing to research on necrotizing pancreatitis. Topping the list is the *Postgraduate Institute of Medical Education and Research* in India, with 25 publications (9.96%), followed by the *University of Amsterdam* in the Netherlands (23 publications, 9.16%) and *Central South University* in China (19 publications, 7.57%). Notably, five of these institutions are located in the Netherlands, while three are based in China.

**Table 3:** List of universities and medical institutions by the number of publications in the field of NP research.

№	Institution	Country	Publication (%)
1	Postgraduate Institute of Medical Education and Research	India	25 (9.96%)
2	University of Amsterdam	Netherlands	23 (9.16%)
3	Central South University	China	19 (7.57%)
4	Indiana University School of Medicine	United States	17 (6.77%)
5	Sichuan University	China	13 (5.18%)
6	St. Antonius Hospital	Netherlands	13 (5.18%)
7	Jeroen Bosch Hospital	Netherlands	11 (4.38%)
8	University Medical Center Utrecht	Netherlands	11 (4.38%)
9	Capital Medical University	China	10 (3.98%)
10	Isala Clinics	Netherlands	8 (3.18%)

**Figure 5:**
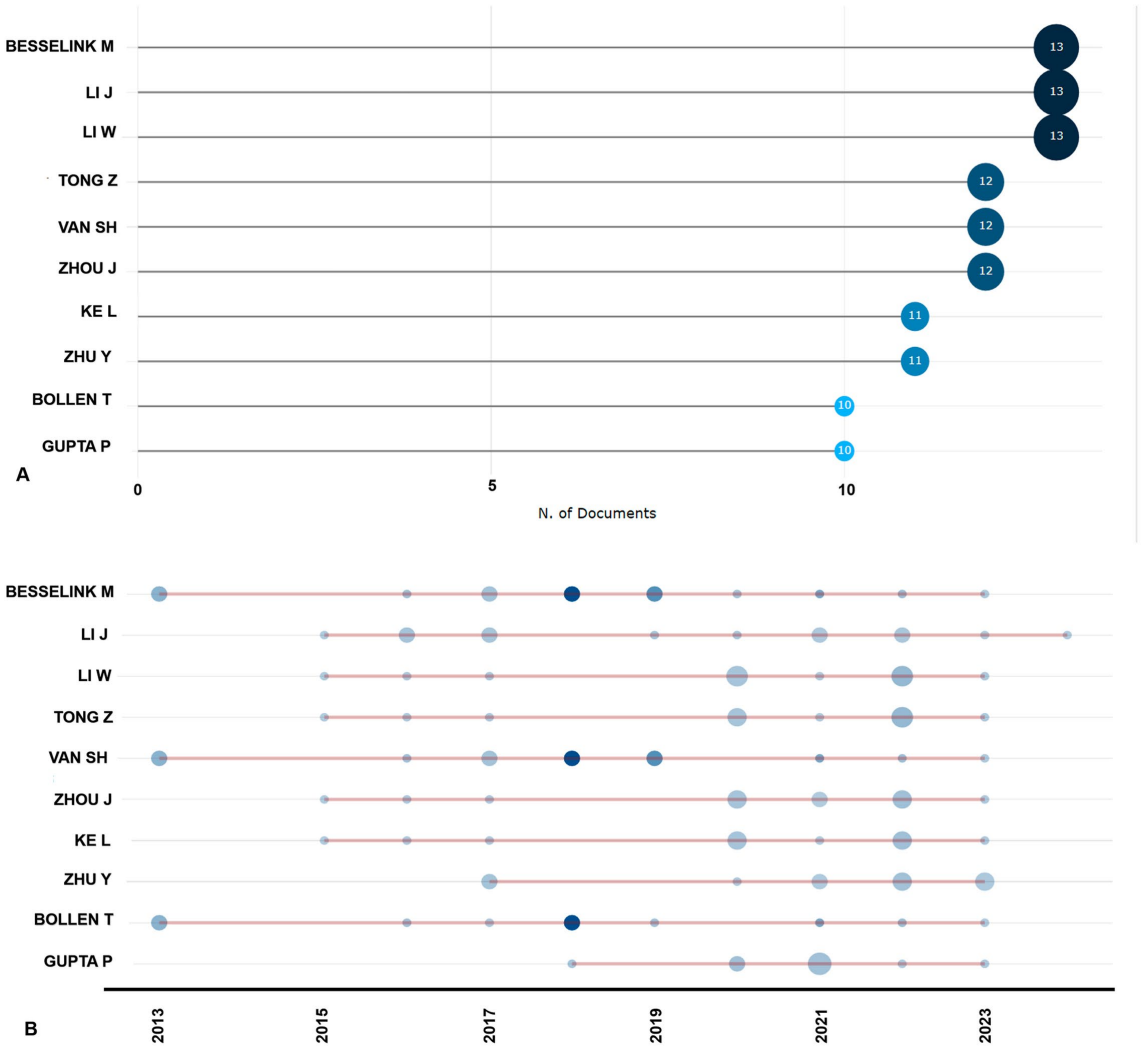
Top 10 most productive authors **(A)**, authors’ production over time **(B)**.

Figure 5A illustrates the most prolific authors in the field, with Besselink M., Li J., and Li W. leading with 13 publications each. They are closely followed by Tong Z., Van S.H., and Zhou J., each contributing 12 publications. Notably, Besselink M. and Bollen T. are notable for their frequent collaborations and high citation counts, particularly in 2018. Gupta P. displayed significant citation activity in 2018 and 2020 but saw a decline by 2022. Conversely, Ke L. reached a peak citation index in 2022, despite low citation counts in 2015 and 2021. Meanwhile, authors Li J., Li W., and Tong Z. exhibit varying citation patterns across years, indicating differences in the temporal impact of their work (Figure 5B).

**Figure 6:**
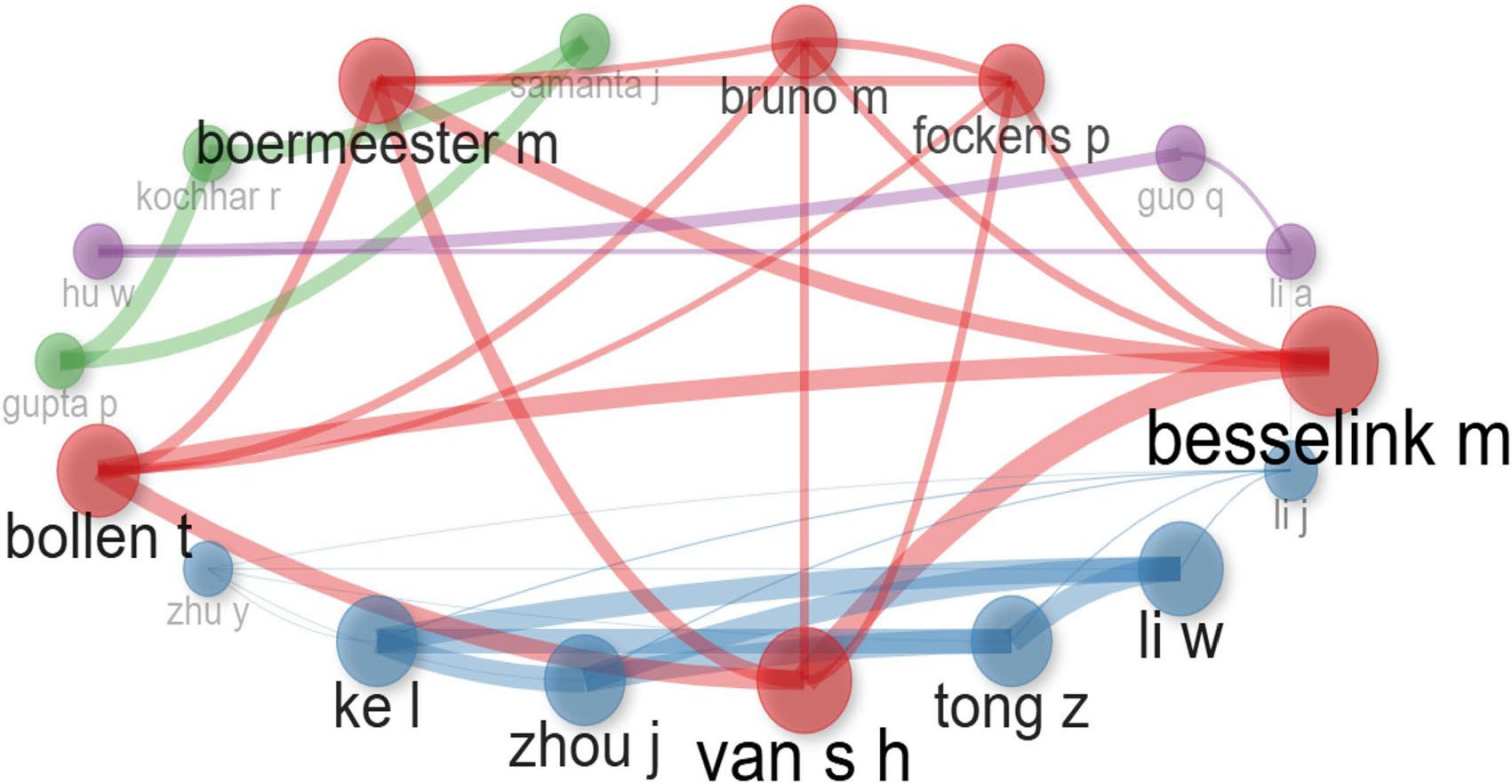
Analysis of co-citation patterns of authors in studies of necrotizing pancreatitis.

Figure 6 illustrates a co-authorship and co-citation network based on cluster analysis. The network is divided into four distinct clusters, differentiated by node color. The *red cluster* features authors such as Besselink M., Boermeester M., and Bollen T., who are tightly interconnected, indicating strong collaborative ties and a high degree of co-authorship within their research area. The *blue cluster*, dominated by authors like Ke L., Zhou J., Tong Z., and Li W., primarily from Chinese institutions, exhibits dense connections, signifying frequent and robust collaborations. This cluster demonstrates the most significant internal cohesion, as reflected by the thick lines connecting its members. The *purple cluster* includes authors such as Hu W. and Guo Q., who display notable connections within the group. While this cluster is smaller and less dense than the red and blue clusters, it highlights a cohesive network among its members. Finally, the *green cluster* consists of authors like Gupta P., Kochhar R., and Samanta, who maintain consistent connections with one another. This cluster represents a distinct group of researchers with aligned thematic interests and a moderate level of collaborative activity.

### 3.6 Authors keywords

**Figure 7:**
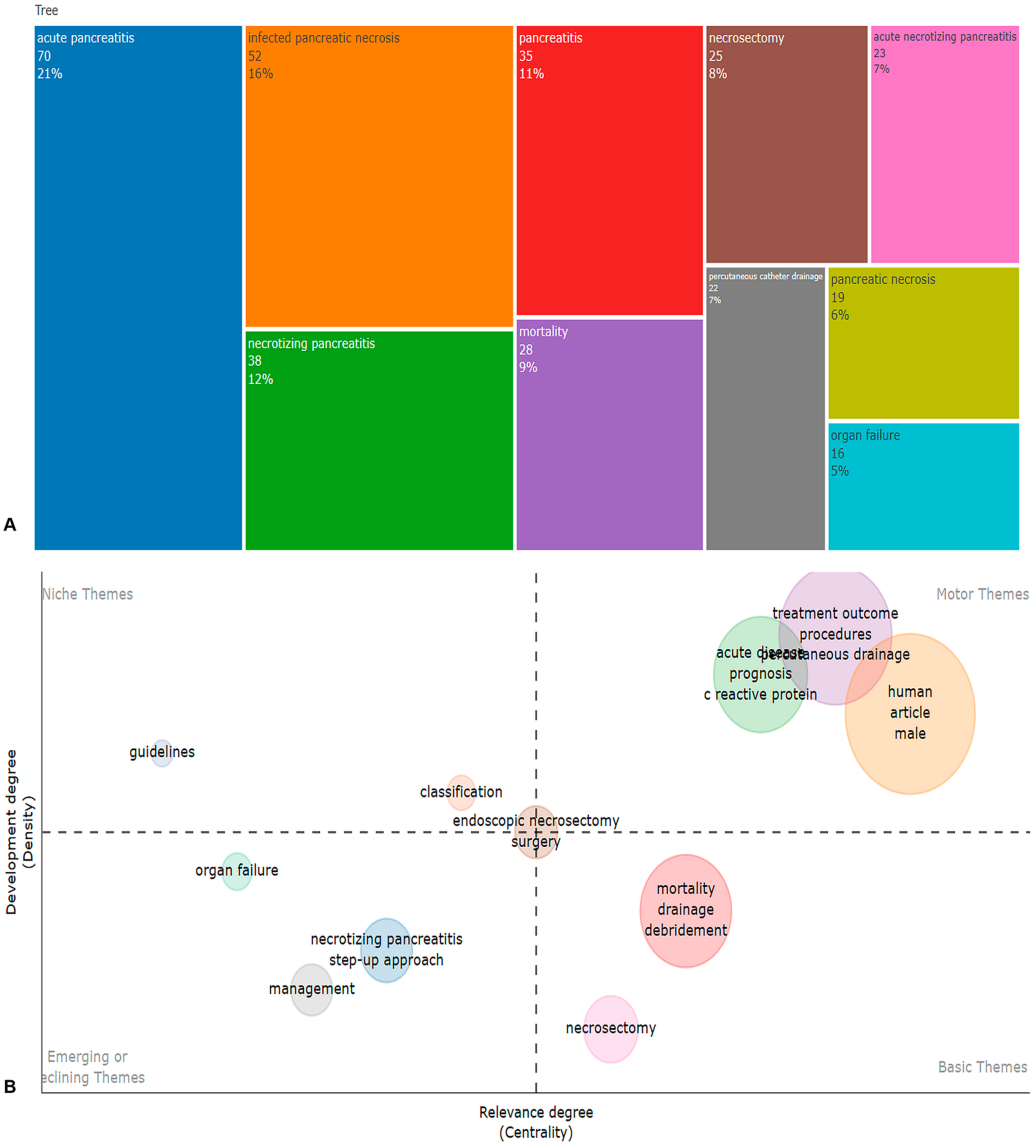
Tree map about frequency of mention of various terms and keywords **(A)**, thematic keyword map **(B)**.

The analysis of frequently occurring keywords reveals the primary research foci in the field of necrotizing pancreatitis. The terms “acute pancreatitis” (70 mentions), “infected pancreatic necrosis” (52 mentions), and “necrotizing pancreatitis” (38 mentions) predominate, indicating a concentrated focus on the severe forms of the disease. Additionally, keywords such as “mortality” (28 mentions) and “necrosectomy” (25 mentions) emphasize the significant role of surgical interventions in managing complications associated with necrotizing pancreatitis. Moreover, minimally invasive techniques, such as “percutaneous catheter drainage” (22 mentions), alongside the management of systemic complications, notably “organ failure” (16 mentions), are identified as critical areas of focus within both clinical applications and ongoing research efforts (Figure 7A).

Figure 7B presents a clustering of key terms related to necrotizing pancreatitis (NP), based on their relevance and level of development. In the “Emerging or Declining Themes” cluster, terms such as organ failure, necrotizing pancreatitis, and management are characterized by low relevance and development, indicating either the early stages of research or decreasing relevance. The “Niche Themes” cluster includes terms like guidelines and classification, which, despite having a high level of development, have low relevance, suggesting their specialized nature. In the “Basic Themes” cluster, terms such as mortality, drainage, and necrosectomy exhibit high relevance but require further research, indicating they are underdeveloped. The “Motor Themes” cluster, which includes terms like treatment outcomes, procedures, prognosis, and C-reactive protein, shows both high relevance and development, emphasizing their central role and well-established research foundation in NP.

## 4 Discussion

The findings of this bibliometric analysis highlight the growing global interest in pancreatic necrosis and associated mortality over the past decade. From 2013 to 2024, there has been a notable increase in the volume of scientific publications on this topic, with a particularly significant growth observed in the last 5 years. This trend underscores the rising recognition of pancreatic necrosis as a critical medical issue, driving extensive research efforts to improve understanding, management, and outcomes. A slight decline in publications in 2023 may be attributed to the reallocation of research funding to other areas, particularly those more directly associated with the pandemic, potentially influencing the volume of publications in subsequent years. By identifying the most productive countries, authors, and institutions, as well as high-impact journals and commonly used keywords, this analysis provides valuable insights into the global research landscape and highlights the collaborative nature of advancements in this field.

Guo Qiang et al. conducted a study revealing that patients with necrotic pancreatitis had a higher mortality rate compared to those with sterile necrosis (33). The primary predictors of mortality identified in the study included bacteremia, advanced age, persistent organ failure during the first week, and pancreatic necrosis. Therefore, a significant portion of the publications analyzed in this study focused on treatment strategies for pancreatic necrosis, with a marked emphasis on minimally invasive approaches. Sandra van Brunschot’s research highlighted that although the endoscopic step-up approach did not surpass the surgical step-up approach in reducing severe complications or mortality in patients with infected necrotizing pancreatitis, it demonstrated advantages such as a reduced incidence of pancreatic fistulas and shorter hospital stays (31). These findings suggest the potential for a paradigm shift toward the endoscopic step-up approach as the preferred treatment method. Similarly, Ji Young Bang’s study reinforced these advantages, finding no significant differences in mortality between the approaches (34). However, patients in the endoscopic group experienced fewer severe complications, no cases of enterocutaneous or pancreatic-cutaneous fistulas, better physical health and quality of life outcomes at 3 months, and significantly lower costs ($75,830 compared to $117,492 for surgical intervention). These findings align with the evidence reviewed by Todd H. Baron in the American Gastroenterological Association (AGA) Institute Clinical Practice Update, which emphasized the importance of individualized and evidence-based management in optimizing outcomes and reducing morbidity in pancreatic necrosis (32). The comprehensive review, commissioned and approved by the AGA Institute Clinical Practice Updates Committee and the AGA Governing Board, was based on 15 best practice recommendations developed by experts in the field. These recommendations integrate insights from key studies and the extensive clinical experience of authors, including leading endoscopists and hepatopancreatobiliary surgeons, to provide guidance for effective management of this challenging condition.

Leading journals that cover a broad spectrum of topics, such as surgery and gastroenterology, have contributed significantly to the research on pancreatic necrosis. Additionally, most of the journals cited in the primary sources were highly influential, falling within the top quartiles (Q1 and Q2). The choice of high-impact, peer-reviewed journals is essential for ensuring the reliability of scientific findings, thus enhancing the quality of the evidence (35). This is particularly crucial, as policymakers and healthcare providers often base their decisions on robust, high-quality evidence (36). Authors consider several factors when choosing a journal for submission, including its impact factor, JCR ranking, open access options, indexing in major databases, and the associated publication fees (37).

In our study, China emerged as the most productive country in the field of pancreatic necrosis research, which aligns with the findings of other bibliometric analyses (29). Furthermore, among the four identified clusters of collaboration networks, the strongest and most prominent was the cluster formed predominantly by Chinese authors. This prominence may be attributed to the stable incidence rate and the high mortality associated with pancreatic necrosis in China, which likely motivated a focused national response to tackle this pressing healthcare issue (2, 14, 15). The strong collaborative efforts within this cluster highlight significant investments in research infrastructure, funding, and the prioritization of healthcare-related studies in the country (38, 39). Another contributing factor is the involvement of the Chinese Acute Pancreatitis Clinical Trials Group (CAPCTG) and the Chinese Critical Care Nutrition Trials Group (CCCNTG) in international randomized controlled trials. This marks a significant step toward integrating into the global research community and enhancing international collaboration in the field (40). Furthermore, China’s leadership in pancreatic necrosis research can be attributed to its strong focus on fostering innovation and creating an enabling environment for scientific advancement. The government prioritizes increased investments in educational funds to develop innovative talent and support the growth of research organizations. Additionally, legislative reforms have been introduced to strengthen intellectual property protection and combat rights violations, ensuring the rights and interests of researchers and stakeholders are safeguarded (41).

Among all universities, Netherlands institutions have proven to be the most prominent, with the University of Amsterdam standing out as a leader. One of the studies conducted by their research group focuses on clinical symptoms and treatment approaches, specifically *Indication and timing for intervention*, and is published in the high-impact journal *The Lancet* (42). Additionally, a multicenter randomized superiority trial was carried out involving patients with infected necrotizing pancreatitis. This trial compared the effects of immediate drainage within 24 h and delayed drainage after the formation of walled-off necrosis. The findings revealed that delayed drainage offered advantages in terms of reduced complication rates and fewer invasive interventions (43). Furthermore, in collaboration with experts specializing in minimally invasive surgery from America, Europe, and Oceania, they played a crucial role in the development of the guidelines for robotic pancreatic surgery (44). Notably, researchers from this university form the largest collaboration cluster (red cluster) dedicated to addressing issues related to pancreatic necrosis and are part of the Dutch Pancreatitis Study Group.

Furthermore, а key factor in the success of the Netherlands in the field of pancreatic necrosis research is the strong national collaboration between hospitals and universities, spanning a wide range of disciplines including surgery, gastroenterology, epidemiology, biostatistics, and radiology. This collaborative framework has facilitated the country’s leadership in citation counts within this area. For instance, in the study by Olaf Bakker et al., five prominent Netherlands institutions played a pivotal role in comparing the efficacy of endoscopic and surgical necrosectomy in patients with infected necrotizing pancreatitis (25). This collaborative effort underscores the importance of national partnerships between leading healthcare institutions and academic centers in achieving exceptional clinical outcomes. Moreover, it highlights the significant impact of such interdisciplinary cooperation in advancing medical research and treatment strategies for complex conditions like pancreatic necrosis.

In the conducted analysis, the most frequent key terms were related to acute *pancreatitis, pancreatic necrosis, necrosectomy*, and *mortality*. These terms highlight significant areas of research focused on the pathogenesis, treatment, and outcomes of pancreatic diseases. Although significant progress has been made in the diagnosis and treatment of acute pancreatitis, pancreatic necrosis continues to pose a major challenge due to its association with a high mortality rate (9) and requires further investigation into timely diagnosis, optimization of treatment (32), and the exploration of novel interventions such as surgical and endoscopic necrosectomy (31). The high mortality rate from infected pancreatic necrosis continues to be a pressing concern, emphasizing the need for alternative treatment methods and the development of more accurate predictive models to improve clinical outcomes.

## 5 Conclusion

In this bibliometric analysis, we aimed to systematically review and evaluate the global literature on the topic of mortality due to pancreatic necrosis. The analysis of the publications enabled us to identify key research trends and directions in the study of this condition. Despite advancements in medical science, the mortality rate associated with pancreatic necrosis remains high. This highlights the continued challenge in effectively addressing complications of acute pancreatitis. Researchers worldwide are actively exploring alternative therapeutic approaches to mitigate these complications and improve patient outcomes. Thus, while progress has been made, there is an ongoing need for innovative treatment strategies to further reduce the morbidity and mortality linked to this severe condition.

## Data Availability

The raw data supporting the conclusions of this article will be made available by the authors, without undue reservation.

## Footnotes

^1^ https://cran.r-project.org/web/packages/bibliometrix/

## References

[1] Lee PJ Papachristou GI. New insights into acute pancreatitis. Nat Rev Gastroenterol Hepatol. (2019) 16:479–96. doi: 10.1038/s41575-019-0158-2 31138897

[2] Iannuzzi JP King JA Leong JH Quan J Windsor JW Tanyingoh D . Global incidence of acute pancreatitis is increasing over time: a systematic review and Meta-analysis. Gastroenterology. (2022) 162:122–34. doi: 10.1053/j.gastro.2021.09.043, PMID: 34571026

[3] Zilio MB Eyff TF Azeredo-Da-Silva ALF Bersch VP Osvaldt AB. A systematic review and meta-analysis of the aetiology of acute pancreatitis. HPB. (2019) 21:259–67. doi: 10.1016/j.hpb.2018.08.003 30249509

[4] Kokosis G Perez A Pappas TN. Surgical management of necrotizing pancreatitis: an overview. World J Gastroenterol. (2014) 20:16106–12. doi: 10.3748/wjg.v20.i43.16106, PMID: 25473162 PMC4239496

[5] Lankisch PG Apte M Banks PA. Acute pancreatitis. Lancet. (2015) 386:85–96. doi: 10.1016/s0140-6736(14)60649-8, PMID: 25616312

[6] Bostancı Ö Battal M Yazıcı P Demir U Alkım C. Management of iatrogenic injuries due to endoscopic sphincterotomy: surgical or conservative approaches. Turk J Surg. (2018) 34:24–7. doi: 10.5152/turkjsurg.2017.3820, PMID: 29756102 PMC5937654

[7] Garg PK Singh VP. Organ failure due to systemic injury in acute pancreatitis. Gastroenterology. (2019) 156:2008–23. doi: 10.1053/j.gastro.2018.12.041, PMID: 30768987 PMC6486861

[8] Sarri G Guo Y Iheanacho I Puelles J. Moderately severe and severe acute pancreatitis: a systematic review of the outcomes in the USA and European Union-5. BMJ Open Gastroenterol. (2019) 6:e000248. doi: 10.1136/bmjgast-2018-000248, PMID: 30899535 PMC6398872

[9] Gliem N Ammer-Herrmenau C Ellenrieder V Neesse A. Management of Severe Acute Pancreatitis: an update. Digestion. (2021) 102:503–7. doi: 10.1159/000506830, PMID: 32422634 PMC8315686

[10] Rashid MU Hussain I Jehanzeb S Ullah W Ali S Jain AG . Pancreatic necrosis: complications and changing trend of treatment. World J Gastrointest Surg. (2019) 11:198–217. doi: 10.4240/wjgs.v11.i4.198, PMID: 31123558 PMC6513789

[11] Wolbrink DRJ Kolwijck E Ten Oever J Horvath KD Bouwense SAW Schouten JA. Management of infected pancreatic necrosis in the intensive care unit: a narrative review. Clin Microbiol Infect. (2020) 26:18–25. doi: 10.1016/j.cmi.2019.06.017, PMID: 31238118

[12] Bugiantella W Rondelli F Boni M Stella P Polistena A Sanguinetti A . Necrotizing pancreatitis: a review of the interventions. Int J Surg. (2016) 28:S163–71. doi: 10.1016/j.ijsu.2015.12.038 26708848

[13] Dellinger EP Tellado JM Soto NE Ashley SW Barie PS Dugernier T . Early antibiotic treatment for severe acute necrotizing pancreatitis: a randomized, double-blind, placebo-controlled study. Ann Surg. (2007) 245:674–83. doi: 10.1097/01.sla.0000250414.09255.84, PMID: 17457158 PMC1877078

[14] Xiao J Quan X Liu F Li W. Comparison of different surgical methods for necrotizing pancreatitis: a Meta-analysis. Front Surg. (2021) 8:723605. doi: 10.3389/fsurg.2021.723605, PMID: 34631782 PMC8493073

[15] Yu L Xie F Luo L Lei Y Huang X Yang X . Clinical characteristics and risk factors of organ failure and death in necrotizing pancreatitis. BMC Gastroenterol. (2023) 23:19. doi: 10.1186/s12876-023-02651-4, PMID: 36658497 PMC9850524

[16] Hamada S Masamune A Kikuta K Hirota M Tsuji I Shimosegawa T. Nationwide epidemiological survey of acute pancreatitis in Japan. Pancreas. (2014) 43:1244–8. doi: 10.1097/mpa.0000000000000200, PMID: 25084001

[17] Türkvatan A Erden A Türkoğlu MA Seçil M Yüce G. Imaging of acute pancreatitis and its complications. Part 2: complications of acute pancreatitis. Diagnostic and interventional. Imaging. (2015) 96:161–9. doi: 10.1016/j.diii.2013.12.018 24703377

[18] Zerem E. Treatment of severe acute pancreatitis and its complications. World J Gastroenterol. (2014) 20:13879–92. doi: 10.3748/wjg.v20.i38.13879, PMID: 25320523 PMC4194569

[19] Freeman M Werner J Santvoort H Baron T Besselink M Windsor J . Interventions for necrotizing pancreatitis summary of a multidisciplinary consensus conference. Pancreas. (2012) 41:1176–94. doi: 10.1097/MPA.0b013e318269c660, PMID: 23086243

[20] Mehta V Kumar R Parkash S Singla S Singh A Chaudhary J . Role of percutaneous catheter drainage as primary treatment of necrotizing pancreatitis. Turk J Gastroenterol. (2019) 30:184–7. doi: 10.5152/tjg.2018.17542, PMID: 30457559 PMC6408166

[21] van Baal MC van Santvoort HC Bollen TL Bakker OJ Besselink MG Gooszen HG. Systematic review of percutaneous catheter drainage as primary treatment for necrotizing pancreatitis. Br J Surg. (2011) 98:18–27. doi: 10.1002/bjs.7304, PMID: 21136562

[22] Tan J Tan H Hu B Ke C Ding X Chen F . Short-term outcomes from a multicenter retrospective study in China comparing laparoscopic and open surgery for the treatment of infected pancreatic necrosis. J Laparoendosc Adv Surg Tech A. (2012) 22:27–33. doi: 10.1089/lap.2011.0248, PMID: 22217005

[23] Mathew MJ Parmar AK Sahu D Reddy PK. Laparoscopic necrosectomy in acute necrotizing pancreatitis: our experience. J Minim Access Surg. (2014) 10:126–31. doi: 10.4103/0972-9941.134875, PMID: 25013328 PMC4083544

[24] Revoredo Rego F Reaño Paredes G De Vinatea De Árdenas J Kometter Barrios F Alfaro Ita S Vereau RJ. Infected necrotizing pancreatitis. Video-assisted retroperitoneal debridement. Medicina (B Aires). (2021) 81:115–8. 33611255

[25] Bakker OJ van Santvoort HC van Brunschot S Geskus RB Besselink MG Bollen TL . Endoscopic transgastric vs surgical necrosectomy for infected necrotizing pancreatitis: a randomized trial. JAMA. (2012) 307:1053–61. doi: 10.1001/jama.2012.276, PMID: 22416101

[26] Xia C Yin H Zhang K Wang Z Yang X Huang H. The global research status and trends of the application of endoscopic ultrasonography in pancreatic tumors over the last decades: a bibliometric study. Front Oncol. (2022) 12:980415. doi: 10.3389/fonc.2022.980415, PMID: 36033532 PMC9411717

[27] Wu K Liu Y Liu L Peng Y Pang H Sun X . Emerging trends and research foci in tumor microenvironment of pancreatic cancer: a bibliometric and visualized study. Front Oncol. (2022) 12:810774. doi: 10.3389/fonc.2022.810774, PMID: 35515122 PMC9063039

[28] Cui M Hu Y You L Liu Q Lita A Wu W . A bibliometric study on pancreatic cystic disease research. J Pancreatol. (2019) 2:43–7. doi: 10.1097/JP9.0000000000000015

[29] Luo X Zhong R Wang X Yang G Jiang X Peng Y . Twenty-year span of global acute pancreatitis trends: a bibliometric analysis. Pancreatology. (2022) 22:356–66. doi: 10.1016/j.pan.2022.01.009, PMID: 35148958

[30] Sun W Huang P Song H Feng D. Bibliometric analysis of acute pancreatitis in web of science database based on CiteSpace software. Medicine (Baltimore). (2020) 99:e23208. doi: 10.1097/md.0000000000023208, PMID: 33285696 PMC7717787

[31] van Brunschot S van Grinsven J van Santvoort HC Bakker OJ Besselink MG Boermeester MA . Endoscopic or surgical step-up approach for infected necrotising pancreatitis: a multicentre randomised trial. Lancet. (2018) 391:51–8. doi: 10.1016/S0140-6736(17)32404-2, PMID: 29108721

[32] Baron TH DiMaio CJ Wang AY Morgan KA. American Gastroenterological Association clinical practice update: management of pancreatic necrosis. Gastroenterology. (2020) 158:67–75.e1. doi: 10.1053/j.gastro.2019.07.064, PMID: 31479658

[33] Guo Q Li A Xia Q Liu X Tian B Mai G . The role of organ failure and infection in necrotizing pancreatitis: a prospective study. Ann Surg. (2014) 259:1201–7. doi: 10.1097/SLA.0000000000000264, PMID: 24169172

[34] Bang JY Arnoletti JP Holt BA Sutton B Hasan MK Navaneethan U . An endoscopic transluminal approach, compared with minimally invasive surgery, reduces complications and costs for patients with necrotizing pancreatitis. Gastroenterology. (2019) 156:1027–40.e3. doi: 10.1053/j.gastro.2018.11.031, PMID: 30452918

[35] Lunny C Neelakant T Chen A Shinger G Stevens A Tasnim S . Bibliometric study of 'overviews of systematic reviews' of health interventions: evaluation of prevalence, citation and journal impact factor. Res Synth Methods. (2022) 13:109–20. doi: 10.1002/jrsm.1530, PMID: 34628727

[36] Tunis SR Stryer DB Clancy CM. Practical clinical trials: increasing the value of clinical research for decision making in clinical and health policy. JAMA. (2003) 290:1624–32. doi: 10.1001/jama.290.12.1624, PMID: 14506122

[37] Wenaas L. Choices of immediate open access and the relationship to journal ranking and publish-and-read deals. Front Res Metr Anal. (2022) 7:943932. doi: 10.3389/frma.2022.943932, PMID: 36339745 PMC9632291

[38] Mao W Li K Zhou J Chen M Ye B Li G . Prediction of infected pancreatic necrosis in acute necrotizing pancreatitis by the modified pancreatitis activity scoring system. United European Gastroenterol J. (2023) 11:69–78. doi: 10.1002/ueg2.12353, PMID: 36579414 PMC9892470

[39] Ke L Zhou J Mao W Chen T Zhu Y Pan X . Immune enhancement in patients with predicted severe acute necrotising pancreatitis: a multicentre double-blind randomised controlled trial. Intensive Care Med. (2022) 48:899–909. doi: 10.1007/s00134-022-06745-7, PMID: 35713670 PMC9205279

[40] Chinese Acute Pancreatitis Clinical Trials Group and the Chinese Critical Care Nutrition Trials Group. Bid farewell to the old year and usher in the new. (2024). Available at: https://capctg.medbit.cn/en/2024/02/08/bid-farewell-to-the-old-year-and-usher-in-the-new/.

[41] Liu L Zhang Y Zhang J Zhang S. Coupling coordination degree of government support, financial support and innovation and its impact on economic development. IEEE Access. (2020) 8:104039–51. doi: 10.1109/ACCESS.2020.2999501

[42] Boxhoorn L Voermans RP Bouwense SA Bruno MJ Verdonk RC Boermeester MA . Acute pancreatitis. Lancet. (2020) 396:726–34. doi: 10.1016/S0140-6736(20)31310-6 32891214

[43] Boxhoorn L van Dijk SM van Grinsven J Verdonk RC Boermeester MA Bollen TL . Immediate versus postponed intervention for infected necrotizing pancreatitis. N Engl J Med. (2021) 385:1372–81. doi: 10.1056/NEJMoa2100826, PMID: 34614330

[44] Liu R Abu Hilal M Besselink MG Hackert T Palanivelu C Zhao Y . International consensus guidelines on robotic pancreatic surgery in 2023. Hepatobiliary Surg Nutr. (2024) 13:89–104. doi: 10.21037/hbsn-23-132, PMID: 38322212 PMC10839730

[45] Trikudanathan G Tawfik P Amateau SK Munigala S Arain M Attam R . Early (<4 Weeks) Versus Standard (≥4 Weeks) Endoscopically Centered Step-Up Interventions for Necrotizing Pancreatitis. Am J Gastroenterol. (2018) 113:1550–8. doi: 10.1038/s41395-018-0232-3 30279466

[46] Trikudanathan G Wolbrink DRJ van Santvoort HC Mallery S Freeman M Besselink MG. Current Concepts in Severe Acute and Necrotizing Pancreatitis: An Evidence-Based Approach. Gastroenterology. (2019) 156:1994–2007.e3. doi: 10.1053/j.gastro.2019.01.269 30776347

